# Individual and school level correlates of moderate to vigorous physical activity among school-children in Germany – a multi-level analysis

**DOI:** 10.1186/s12889-015-1715-4

**Published:** 2015-04-17

**Authors:** Fabian Czerwinski, Emily Finne, Petra Kolip, Jens Bucksch, the HBSC Study Group Germany

**Affiliations:** Bielefeld University, School of Public Health, PO Box 100131, Bielefeld, D-33501 Germany

**Keywords:** (Moderate-to-vigorous) physical activity, Adolescents, Correlates, School, Environment

## Abstract

**Background:**

Young people spend half of their days in school, but evidence concerning the influence of school environment on the physical activity (PA) of pupils is still inconsistent. A better understanding of potential correlates of PA on the school-level and their possible interaction with individual aspects is needed to improve the development of more effective interventions.

**Methods:**

We used data from the 2009/10 German Health Behaviour in School-aged Children study (HBSC)-sample (n=5,005 students aged 11–15 years) including self-reported moderate to vigorous intensity PA as well as a variety of biological, demographic and behavioral correlates and matched them with school-level data from the national school principals’ HBSC questionnaire. We analyzed the associations of individual- and school-level correlates with MVPA by gender-specific multi-level regression.

**Results:**

Only a small share of the overall variation in student’s PA was attributable to the school-level. Consequently, the associations of individual-level correlates with PA were stronger than those of the school-level. Our analysis revealed significant associations of individual-level (i.e. age, consumption of softdrinks, overweight) as well as school-level correlates (i.e. the availability of a football ground and a swimming pool) with MVPA. We also observed some gender-specific findings especially for the school level correlates. Cross-level interactions between individual- and school-level were not apparent.

**Conclusions:**

Our findings indicate the usefulness of applying an ecological framework to understand and explain complex health behaviors like PA. As we found gender-specific association it might be important to acknowledge that boys and girls have specific needs to be more physically active. Further research should also take other features/elements of the school environment and neighborhood as well as socio-cognitive correlates into account to advance the field.

## Background

There is a large body of literature highlighting a positive relationship between physical activity (PA) and adolescents’ health including physical, mental and social indicators [1,2]. From a health perspective it is recommended for children and adolescents to be physically active for at least 60 minutes with moderate-to-vigorous-intensity (MVPA) [3,4]. However, pooled data of Health Behaviour in School-aged Children study (HBSC) including 41 countries stressed that only 19% of the girls and 28% of the boys at the age of 11 met this health-related PA recommendation. Furthermore the prevalence decreases with increasing age during adolescence. Taking the German sample out of the pooled HBSC-data the prevalence decreased from 19.8% to 8.6% between the age of 11 to 15 in girls and from 24.9% to 13.6% in boys, respectively [5]. Data from another representative German study reported that only 8.3% (girls) and 9.4% (boys) within the age range 11 to 13 years fulfilled the health-related PA recommendation [6].

This alarmingly low prevalence of MVPA underlines the need for a deeper understanding of the determinants of daily MVPA among children and adolescents as a prerequisite to develop physical activity promotion interventions [7]. The scientific discussion surrounding correlates and determinants of health behaviors has been dominated by individual-centered theories and intraindividual variables at least until the WHO adopted the Ottawa-Charta in 1986. Since the late 1980s, the focus has been widened up to more multi-level approaches that also integrate factors at the environmental level to explain health-related behaviors [8]. Within the last decade, several PA-specific social-ecological models have been proposed and provide a more holistic and concrete framework for a broader understanding of the potential determinants and correlates of PA [9,10].

As children usually spend about half of the day at school, more research should be directed towards the understanding of potential determinants of MVPA in schools [11]. Therefore the primary focus of our paper is on the behavior setting school (excluding aspects of commuting to school) and on the intrapersonal level and their interaction to explain MVPA levels. The use and analysis of matched data from the school level as well as from the individual level is the specific strength of our approach, as most previous studies analyzed data from only one of these levels. Furthermore we use a representative German sample of schools. Therefore we will summarize recent key findings on the intrapersonal level as well as the school level in the following. We acknowledge that there are also social level correlates of PA [12,13], but this is beyond our scope.

At the intrapersonal level systematic reviews [7,14,15] conclude that demographic and biological variables (e.g. age, sex, migration background), psychological variables (e.g. self-efficacy, motivation, self-rated-health) as well as behavioral variables (as spent time outside, healthy nutrition) contribute to explain the PA of children and adolescents.

At the school level recent findings highlighted that PA is associated with a range of conceptual, organizational and structural characteristics forming the behavior setting of children and adolescents during the school day. In general children younger than 13 years were more active in schools with a PA-related concept [16,17]. The number of physically active classmates was positively associated with boys’ and girls’ PA, whereas schools with varsity sport programs have been found to come along with lower PA-prevalence of boys [18]. Children at public schools were more active than at private schools and the teacher’s educational skills regarding PA and sports were consistently reported to be associated with higher PA-rates of the children [17].

For 13- to 15-years old adolescents, positive associations were reported between overall PA and the number of weekly physical education lessons at school as well as between the participation in school sports and overall PA [19]. Consistently, other authors found the number and quality of the weekly physical education lessons to be positively correlated with the overall PA of adolescent boys and girls [20,21]. Concerning structural aspects in schools, positive associations were reported for the availability of PA-equipment (e.g. balls) and the accessibility of school-fields and yards after school [20,22-24]. Bocarro and colleagues found PA in general and the use of PA-equipment and -fields to be gender-specific: for example, coeducational physical education lessons tend to be widely dominated by boys, thus inhibiting girls from a more active participation [18]. Another study found that children were most active in school environments with higher levels of supervision and a greater variety in improved courts or playgrounds; whereas boys’ PA was closely associated with the availability of supervised outdoor courts and fields, girls were reported to prefer supervised indoor PA [25].

In summary, the small but growing body of evidence in terms of the association between school environment and PA of pupils is still inconsistent [17] and only few studies analyzed correlates from different levels of influence with a school framework. The following analysis aims to quantify the associations of several school-level as well as intrapersonal variables on MVPA among 11- to 15-years old German school children and to examine interactions between school-level and intrapersonal variables. Since PA correlates and PA levels differ between boys and girls we run gender-specific analyses [5,18,19].

## Methods

### Participants and procedures

The data of this study are based on the 2009/10 German Health Behavior in School-aged Children (HBSC) Survey. HBSC is an international health survey in students aged 11 to 15 years old. It is conducted under the auspices of the WHO since 1982 every four years. Detailed information on the international study can be found elsewhere [26]. In brief, every participating country collects self-reported data among students using an internationally consented questionnaire. The questionnaire covers a wide range of topics including health behaviors, several aspects of subjective health and well-being, lifestyle and social networks of students, indicators of the school climate and demographics. In Germany, an additional questionnaire was distributed to each school principal which included items about school demographics, school policy concerning health aspects, and the infrastructure of the school that may influence the PA-levels of the individuals.

Pupils were recruited by a random sampling procedure of schools stratified by federal state and type of school. The sampling approach is standardized by the international protocol [27]. In 2009/10 the HBSC survey was administered to a representative sample of n=5.005 pupils (51.47% girls) from 277 schools across Germany. The response rate at school level was 48%, at students’ level it was 86%.

With respect to human subjects, written informed consent to participate was obtained from parents (or guardians) and from individual students. Ethical approval for the German HBSC was provided by the medical council on behalf of the University of Hamburg. Both data sets (the student’s data and the school’s data) were matched by a unique school code. Due to missing values on either the student’s or the school level, n=128 cases dropped out and n=4,877 students (51.47% girls) from 273 schools remained in the matched dataset.

### Measures

*MVPA (Outcome):* MVPA was assessed by asking “On how many days in the past week were you physically active for 60 minutes or more”? Physical activity was defined as “any activity that increases your heart rate and makes you get out of breath some of the time” with examples of such activities. Response categories were: “0 days”, “1”, “2”, etc. up to “7 days”. This question was developed by Prochaska, Sallis & Long [28] and originally included a further question asking for “days in a typical week”. For the original version good test-retest reliability [28,29] and validity in terms of substantial correlations with accelerometers was shown [28,30]. Due to the limited space in the HBSC survey and a high correlation between activity “in the past 7 days” and “a typical week”, only the “past week” item was used [31].

*Potential correlates:* Correlates in the present analysis include information from both the student’s and the principal’s questionnaires. The information gathered from the student’s questionnaire ranged from socio-demographic correlates age and socio-economic status (SES) to the self-rated health and well-being. The migration background of the students’ parents was assessed by a question if one or both parents were born in another country and migrated to Germany – this information was used to construct a new variable with three categories (no migration background, one parent, both parents). SES was measured by applying the validated Family Affluence Scale (FAS; see [32]); the seven-point FAS-score was divided into three groups representing students from families with low, middle and high affluence. The self-rated health status was determined by using a five-point-scale ranging from “very poor” to “very good”. The values were dichotomized into better (“good or very good”) and poorer (“fair, poor and very poor”) health.

Additionally, the student’s scores on several behavioral correlates were integrated: while the question on “having daily breakfast during the week” was administered as a yes/no-dummy and remained at the original binary scale, other aspects of a healthy nutrition (consumption of fruits and vegetables; consumption of soft-drinks) were measured by asking for the number of days the respective behavior occurs in a typical week. The data on fruits, vegetables and soft-drinks were merged into dummies by median-splits, thus indicating whether or not the students reported the behavior for five or more days/week.

Furthermore, the data included as biological correlates self-reported height and weight, which were used to calculate the individual body mass index indicating underweight as well as overweight and obesity according to the gender- and age-specific reference percentiles developed for children in Germany [33]. We constructed a categorical variable with three levels: BMI-scores between the 10th and 90th percentiles of the reference values were coded as being in the healthy range, we supposed scores below the 10th percentile to indicate underweight and scores above the 90th percentile to indicate overweight or obesity.

In terms of perceived environment data from three questions concerning the degree of perceived deprivation of the neighborhood (ranging three-points from “no problems” to “lots of problems” and asking for “gangs causing trouble“, “litter, broken glass or rubbish lying around” and “housing problems in the living area”) were also taken into account as dummy variables indicating “some or lots of problems”. For more details on the variables and scales depicted here see [5].

School-level correlates from the school administrator’s questionnaire included information on size (numbers of students and teachers) and type (ranging from “Hauptschule”, “Realschule”, “Gesamtschule” up to “Gymnasium” and indicating an increase in educational attainment) of the school, as well as conducting health promotion activities in different areas (diet and nutrition, sports and physical activity etc. – coded as overall-dummy yes/no). The principals have also been asked about PA-related infrastructure of the school, each question as a simple binary variable: availability of a sports hall, a swimming pool, a soccer field, a school yard offering several opportunities to be physically active during breaks or after school, a skating area, a sports field and areas where students may relax and calm. Additionally, the after-school accessibility of these offers was asked for in binary form (yes/no).

Across all age groups, nearly half of the students (43.2%) attended a “gymnasium”, this school type may be categorized as the German equivalent of an academic high school which has been shown to be associated with differing rates of PA [16,17]. Therefore the school types were dichotomized into “gymnasium” and “other” school types, the latter indicating a lower educational attainment. Table 1 shows the descriptive statistics of the sample.

**Table 1:** **Descriptive characteristics of the sample (%, weighted counts)**

**Age**	**Sex**	**School type “gymnasium”**	**Family Affluence Scale**			Mean days of weekly MVPA*	**Total n**	
			**low**	**med**	**high**			
11	Boys	42.8%	6.9%	35.4%	57.7%	4.62	842	1650
	Girls	41.3%	11.0%	39.0%	50.0%	4.32	808	
13	Boys	42.6%	5.2%	33.3%	61.5%	4.41	789	1591
	Girls	45.5%	8.2%	38.2%	53.5%	3.99	802	
15	Boys	42.4%	7.1%	36.9%	56.1%	3.96	715	1588
	Girls	47.1%	8.2%	38.2%	53.7%	3.47	873	
Total	Boys	42.6%	6.4%	35.1%	58.5%	4.36	2346	4829
	Girls	44.7%	9.1%	38.4%	52.5%	3.91	2483	
	Both	43.7%	7.8%	36.9%	55.4%	4.13	4829	

*moderate-to-vigorous physical activity.

### Data screening

All independent variables were evaluated for multicollinearity separately for the individual and the school level variables. We did not observe any problems with multicollinearity since all bivariate correlations were inconspicuous.

The distribution of the outcome variable was checked for assumptions of normality by analyzing the distribution graphically and statistically. Although kurtosis and skewness revealed only small values (k: 2.11; s: −0.06), tests conducted to check for normal distribution led to a rejection of the hypothesized normal distribution (p-values <.01). Different transformations of the scale did not improve the distribution. Because the reported significant deviation from normality is supposed to be mainly attributable to the large sample size, we remained at the original scale of the PA-variable.

### Analysis strategy

The data collected from the students are clustered within schools. Two-level models with variances at the individual and the school-level were applied to account for the clustered structure of the data and to produce accurate estimates of individual standard errors. Following the modeling strategy described by Hox [34], the first step consisted of computing the empty models and the intraclass correlation (ICC) for both sexes. The ICC indicates the share of variance that is attributable to the contextual (school) level [35]. The second step consisted of computing bivariate models for each independent variable and the outcome, separately for girls and for boys. Two variables asking for problems with gangs and with litter in the neighborhood, respectively, and two questions regarding the household composition showed no associations with PA in bivariate analysis and were therefore excluded. In step three, two Random-Intercept-Models were fitted both for girls and for boys. The first models (M1 & M2) only included independent variables on the individual level, whereas M3 & M4 also included covariates on the school level. In step four, we further tested whether the model fit may be improved when the slopes are allowed to vary between the schools [34].

For the following analyses all cases with one or more missing values were excluded, resulting in n=1,464 girls from 164 schools across Germany and n=1,295 boys from 154 schools for further analyses. Excluded cases showed equally distributions of sex and FAS, but a lower share of students attending a “Gymnasium” (41.0% vs. 43.7%) and more students reporting one or both parents were born in a foreign country (29.5% vs. 26.4%). With regard to the outcome, cases excluded due to missing values in at least one of the covariates reported a slightly lower level of MVPA (4.06 days vs. 4.13 days). Statistical analyses were conducted with Stata™ 11.2 using the xtmixed-command that provides robust standard errors.

## Results

As depicted in Table 1, the average number of days students engage in at least one hour of MVPA each week decreases with increasing age. This decline is steeper for the girls who showed lower prevalence at all ages compared to boys.

Table 2 shows the results of the empty models, decomposing the variances into the individual level (students) and the school level and giving the ICC-values. In our case, the ICC=0.0243 means that only 2.4% of the overall variance of PA is at the school level. However, the Likelihood-Ratio-Test is significant and indicates that applying a multilevel statistical approach is justified.

**Table 2:** **Results of the empty models**

	**Girls**	**Boys**	**Overall**
V_1_: Variance at level 1 (students)	3.505327	3.681997	3.637761
V_2_: Variance at level 2 (schools)	.0791812	.0910364	.0905945
ICC^a^ (V_2_/ V_1_ + V_2_)	.0221 (2.21%)	.0241 (2.41%)	.0243 (2.43%)
LR-Test^b^ (chi^2^; p)	7.25; p=.007	5.97; p=.015	21.49; p<.001

a: ICC= Intra-Class-Coefficient.

b: LR-Test= Likelihood-Ratio-Test.

Table 3 shows the results of the estimated multivariate regression models as unstandardized regression coefficients indicating the association of the respective variable on the number of days per week with reported MVPA. Due to the relatively low share of school-level variance, the results of the estimated regression coefficients in the multivariate models (see Table 3) including school-level variables (M3 and M4) remained quite stable in comparison to the models focusing on individual variables only (M1 and M2). Age is negatively associated with PA and a migration background of both parents was associated with lower PA only for girls, whereas family affluence revealed no significant effect on PA of both sexes. In case of self-rated health was to be quite good, girls and boys reported higher PA compared to a poorer self-rated health. Daily breakfast showed no association with PA, but a frequent consumption (5 days/week or more) of fruits and vegetables was strongly associated with increased PA-rates for girls as well as for boys. A high frequency of softdrink-consumption went along with a higher PA for boys (.34).

**Table 3:** **Results of gender-specific multivariate regression models predicting MVPA: unstandardized regression coefficients from two level models including predictors at the individual level (students) and school/contextual level (school administrators)**

	**Variable**	**Girls**		**Boys**	
	**Model**	**M1**	**M3**	**M2**	**M4**
Level	N (students; schools)	(1464; 164)	(1295; 154)		
	Constant	3.47	3.67	3.56	3.49
Demographic	Age 11	Ref.	Ref.	Ref.	Ref.
	Age 13	-.24*	-.23	-.26*	-.24
	Age 15	-.83**	-.82**	-.68**	-.66**
	Migrational background of parents				
	none	Ref.	Ref.	Ref.	Ref.
	one parent	-.04	-.02	.07	.06
	both parents	-.38**	-.38**	.21	.17
	FAS low	Ref.	Ref.	Ref.	Ref.
	FAS medium	-.13	-.12	.22	.20
	FAS high	.29	.29	.24	.23
Behavioral	Self-rated health >=good	.50**	.54**	.53**	.55**
	Daily breakfast	.02	.05	-.01	.01
	Fruit consumption >= 5 d/week	.49**	.51**	.64**	.64**
	Vegetables consump. >= 5 d/week	.48**	.50**	.40**	.40**
	Softdrink consumption >= 5 d/week	-.09	-.11	.35**	.34**
Biological	BMI – healthy range	Ref.	Ref.	Ref.	Ref.
	Underweight	-.00	-.01	.06	.02
	Overweight /Obesity	-.28*	-.31**	-.49**	-.49**
Environmental	Perceived Living Area: housing problems (ref.: “no”)	-.30*	-.31*	-.07	-.06
	School type “Gymnasium” vs. other (ref: “other”)		-.23*		-.09
	Focus on health (general)^a^		-.03		.05
	Sport as area of health-promotion		-.31*		-.17
	Swimming pool		.03		.28*
	Football ground		.30*		.09
	Activity-enhancing schoolyard		.08		.19
	Playground		-.07		-.02
	Skater area		-.17		.21
	Sporting ground		-.09		.25
	Track		-.05		-.30
	Relaxing room		-.07		-.15
	Structures accessible after school		-.03		-.05
	R^2^ (proportional error-reduction)	12.43%	13.69%	13.32%	14.30%
	LR-Test^b^ (M3 vs. M1 & M4 vs. M2)	16.55 (p=.17)		11.73 (p=.47)	

*p<.05 **p<.01 Significant regression coefficients are in bold fonts.

a: the reference category is “no” for all following variables.

b: Likelihood-Ratio-Test.

M1: Model with individual-level variables only for girls.

M2: Model with individual-level variables only for boys.

M3: Model with individual-level and school-level variables for girls.

M4: Model with individual-level and school-level variables for boys.

Students whose BMI indicated underweight did not differ in their PA compared to normal weight children. Overweight and obesity were associated with significant lower PA-rates in girls (−.31) and even more in boys (−.49). Girls perceiving problems with run-down houses in their living-area also significantly reported lower PA (−.31), whereas the PA of the boys revealed no significant association with any correlates of the perceived neighborhood environment.

Regarding variables at the school level, girls attending a “Gymnasium” showed a significant lower level of PA compared with other school types. Furthermore, female students from schools with a health promotion concept focusing on sports/exercise reported lower days of MVPA. Girls attending schools with a football ground had significantly more days of MVPA than girls from schools without such a place. Regarding male students, only the presence of a swimming pool was significantly positive associated with PA. None of the other school-level factors was statistically significant.

The proportional error-reductions of the random-intercept-models compared to the empty models were 12.43% (individual factors only) and 13.69% (individual and school-level factors) for girls and 13.32% and 14.30% for boys, respectively. Regarding the quite low ICC-values (2.21% and 2.41%), the error reduction induced by the school-level variables can be described as acceptable. In contrast, the Likelihood-Ratio-Test indicated no significant improvement of the model fit by integrating the school level predictors (p=.17 and p=.47, respectively).

The random-slope-models were successively built by adding one such random term for each variable while controlling for the other variables. Table 4 shows the chi^2^-values of the Likelihood Ratio-Tests (comparing the Log-Likelihood of each random-slope-model to the Log-Likelihood of the respective fixed-slope-model) and the corresponding p-values for each variable used to fit a random-slope model: The introduction of differential effects between the schools did not improve the overall model-fit (see Table 4). That means the associations between the correlates and the outcome do not differ significantly between schools or, in other words: it is not of any (statistically meaningful) relevance which school is attended by a student with a specific combination of covariates. Consequently we did not test for cross-level interaction effects since we found no indication of significant associations in random slopes. This means that no variation can by explained.

**Table 4:** **Random-slope-models**

	**Girls**	**Boys**
N (students; schools)	(1464; 164)	(1295; 154)
Variable	(LR-Test^a^; p)	
Age	4.05 (0.13)	0.00 (1)
BMI	0.69 (0.71)	- ^b^
Migration background parent(s)	0.10 (0.95)	- ^b^
Self-rated health >=good	1.28 (0.53)	- ^b^
Fruit consumption >= 5 d/week	0.04 (0.98)	0.72 (0.70)
Vegetables consump. >= 5 d/week	- ^b^	- ^b^
Softdrink consumption >= 5 d/week	- ^c^	2.12 (0.35)
Living Area: Run-down houses	0.05 (0.97)	- ^c^

a: Likelihood-Ratio-Test.

b: mixed models not nested.

c: not tested due to former results (see Table 3).

## Discussion

Our findings highlight associations of both individual and school-level correlates with days of a minimum of 60 min of MVPA among 11 to 15 years old German school children. Whereas several individual variables (ranging from socio-demographics to biological and behavioral correlates) revealed a strong association with MVPA, the contextual school-level variables explained only a small amount of MVPA variance. Cross-level interactions between individual- and school-level were not relevant.

We start to discuss our findings in detail with respect to the individual level correlates: In line with a broad range of recent findings, boys showed a higher prevalence of MVPA than girls across all age groups [7,19]. Data from both girls and boys showed a significant decrease in MVPA when getting older. Overall, this age-related prevalence shift is consistent with most other studies [36] as well as with other surveys from Germany [6]. None of the associations of the FAS-scores with the mean MVPA-days reached statistical significance, which is consistent with the majority of findings across national [37] and international studies [38]. The reported studies showed no uniform pattern but varying associations between SES and PA, mainly attributable to the wide range of differing operationalizations of SES and PA across publications and to the kind of covariates adjusted for in the respective analyses. However, we used the MVPA-screening measure as a proxy measure for overall PA including all PA domains (e.g. home, leisure, transport). Different studies have also shown that only the leisure time domain is positively related to a higher SES [39].

Our analyses revealed a gender-specific effect of a migration background of both parents: whereas boys from immigrant families showed only slightly higher prevalence of MVPA than the German reference group, girls from immigrant families reported significantly lower MVPA than girls from the reference group. Research findings concerning the association between ethnic origins and PA are somewhat inconsistent [7]. As most of the data reported in these and other studies [40,41] stem from other cultural backgrounds, the results are hardly comparable to our German data. It is likely, that specific aspects of ethnic minorities living in Germany (e.g. religious or cultural concerns) inhibit girls from being more physically active in their leisure time [42]. As stated for the association between SES and PA it might also be the case that especially leisure time PA but not household or transport-related PA is negatively associated with migration background.

Concerning behavioral correlates and beginning with nutritional aspects, daily breakfast showed no significant associations with MVPA of neither girls nor boys. This finding is consistent with a British study in adolescents [43] and an international comparative study, which reported a positive association between daily breakfast and MVPA for children in most of the 41 countries investigated, but not for children and adolescents in Germany [44]. In contrast, a high intake of fruits and vegetables was strongly associated with higher MVPA in both sexes in our sample. A cross-sectional study of US high-school students also reported higher consumption of fruits and vegetables among the physically high active students [45]. More recent research on the association between fruit and vegetable eating patterns and PA is surprisingly lacking, since there is growing evidence for the clustering of several risk behaviors (including unhealthy diet) in youth [46]. The same is true concerning our finding of a strong positive association between the frequent consumption of soft-drinks and MVPA in boys, which has been linked to an increase in BMI [47] but not yet directly to adolescents’ PA. The positive association between self-rated health (rated “good” or “very good”) and MVPA for both girls and boys in our study is consistent with other recent findings for adolescents [48] as well as for adults [49].

Increased BMI indicating overweight or obesity were negatively correlated with the number of MVPA-days/week of both girls and boys, whereas underweight was not associated with the MVPA of the adolescents in our sample. Due to the cross-sectional design of our study our finding is not able to substantially contribute to clarify causal relationships. In fact, most of the reviews during the last decade reported inconsistent associations between BMI and PA [7]. This lack of distinct findings may be due to the complex interactions und clustering between several health-behaviors and the respective outcomes [46] as well as a possible bi-directional relationship between overweight and PA [50].

We further found a negative association between run-down houses in the neighborhood and the MVPA of girls, which is partly consistent with the results of a review conducted by Limstrand [51], who reported positive correlations between both the aesthetics and the condition of the built environment and young people’s use of sports facilities. Another review found related aspects of local deprivation (e.g., unattended dogs) to have an inhibiting effect on the PA of youth [22]. The gender difference in our results may be due to differences in parents’ perceived risk, which tends to be more distinct for girls than for boys [52]. Other reviews concerning correlates of PA beyond the individual-level tend to focus on familial and social factors [19,41] or on differing aspects of the student’s neighborhood such as traffic volume, walkability, land-use mix and residential density [53].

Concerning school-level correlates, the finding of lower MVPA in girls attending a “Gymnasium” than a school-type with lower educational attainment is a somewhat novel result, as it hasn’t been analyzed until recently in a comparable manner. Concerning the school-type in general, a positive association between high-school (vs. vocational/alternative schools) and adolescents PA has been reported from the US, while for younger children the attendance of a public (vs. private) school was positively associated with more PA [16]. However, studies examining specific aspects of the different school-types in Germany are lacking. Our finding may be partly due to the higher after-school workload of pupils attending a Gymnasium and may be partly explained by girls’ higher performance-orientation, which itself is linked to gender differences in motivation and personality [54]. These differences may discourage girls more than boys from being physically active. The negative association between students MVPA and their school emphasizing “sport as an area of health promotion” (with a significant magnitude of effect for girls only) is similarly lacking comparable research. Our findings may be attributable to an inverse causal relationship between these two constructs: schools with especially low prevalence of girls’ MVPA may have initiated such health promoting activities, but there association remained below the MVPA-threshold of 60 min/day. Alternatively, it can be hypothesized that these activities are dominated by boys – this phenomenon has been reported for a variety of coeducational physical activity programs (e.g. by [18]). Despite some findings about null associations between neither “school support”, “teacher support” nor “instruction on health benefits” and the students’ PA [24] provide us with some hints concerning the inconsistency of results in this specific area of research, more research is needed to elucidate our findings.

We further found gender-specific associations between sport-facilities on the school ground and MVPA: while girls reported significantly higher MVPA when attending a school that provides a football ground, boys showed significantly higher MVPA when their school has an own swimming-pool. These results are in line with international findings linking higher PA to the availability of activity-related equipment and the accessibility of permanent activity structures (e.g., playgrounds, courts etc.) beside the school yard [20,22,23]. The gender-specificity of these findings may be attributable to gender differences in using available structures and opportunities [25], e.g. it might be one hypothesis that boys play football even without a specific field, but girls need a more structured and supervised infrastructure and therefore show a direct benefit due to a school’s football ground. However, none of the other structural factors revealed a significant association with students’ MVPA, which is not too surprising, as other authors judged the evidence concerning the positive effect of both school’s equipment and structures as inconsistent [17].

Our results underline the importance of using an ecological framework to understand and explain variation in the PA of adolescents because correlates of the individual as well as the contextual school level were apparent in our study. When comparing the effects of individual and contextual variables on the school level, it becomes clear that the vast majority of outcome variance is explained by individual variables what is backed by the current literature [55,56]. Nevertheless, specific characteristics of the school can contribute to explain a distinct share of variation in students’ MVPA. Therefore, these results give important advice regarding health-promoting activities of the schools, with a special emphasis on school structures that may increase the PA of the students [57]. The need to deeply understand school-level correlates can lead to the development of more effective interventions, since whole populations (of students) might be reached [58,59]. We did not test for cross-level interactions between individual and school-level correlates, since we failed to detect a significant reduction of the model’s Likelihood by adding random slopes. This is probably due to the fact, that important socio-cognitive correlates as self-efficacy, intention, or attitude [7,19] were not available but these might be more important to identify cross-level interactions. The widespread utilization of validated measures on contextual levels could also strengthen the analysis of such cross-level interactions. However, these theoretical aspects may be further adapted to a holistic framework that describes how the school level impacts on student health with respect to the differential influences of this environment [60].

A few limitations of the current study should be acknowledged. The relatively low ICC-values mirror the individual scope of the HBSC-study and the use of a non-validated instrument for the principals’ questionnaire [27] – research with an a-priori focus on contextual data (e.g. [61]) may have applied more sophisticated techniques and methods. In general, PA seems to be less context-sensitive than other health behaviors (e.g. dental health or tobacco use, see [55]). PA was assessed using a self-report measure. Using an objective measurement technique could improve the overall validity of the analysis [62]. In addition, PA was assessed by using the threshold of at least 1 hour/day MVPA derived from international recommendations. Thus, context-specific associations that may exist below this health-enhancing threshold remain unclear. Furthermore, a psychological fallacy [63] may undermine our results. That is, important information on the school level (e.g., regarding the quality and amount of physical education) wasn’t assessed, despite offering potential to explain PA-variation, as outlined above and elsewhere [21]. We didn’t exclude schools with only few participating students to avoid a further loss of cases (that was caused by missing values on the covariates) within our analyses. This maybe led to estimates with restricted validity concerning the school-level variables, as a cluster-size of 10 or even less individuals is mentioned as problematically [34]. Therefore, we re-analyzed the sample including only schools with a minimum of 10 students (data not shown). In general, we observed only slight differences in regression coefficients for boys and girls that did not alter our main conclusions; so we decided to use as many schools as possible. Finally, we didn’t use corrected p-values for multiple testing due to the explorative character of our analyses, so some of our significant findings may be due to chance [64].

## Conclusions

To conclude, our analyses revealed several associations between school-level variables and MVPA-rates of 11-15-year old children and adolescents in Germany. Therefore interventions in schools should benefit of using a socio-ecological perspective. In addition our gender-specific findings also indicate that a gender-sensitive approach in terms of individual as well as school level correlates should be focused when interventions will be designed. However, the associations with individual correlates were stronger and explained more of the variance in MVPA. Further research should also take other aspects of the school environment and neighborhood into account, as evidence on the influence of such contextual factors on the health behavior of students has become stronger throughout the last years [17,24]. To improve the understanding of causal pathways, the increased use of longitudinal and experimental data is essential to further advances in this field [7] and should ideally be complemented by theoretical considerations on the development of behavior- and environment-specific ecological models [65].

## Abbreviations

BMI: Body-Mass-Index
ICC: Intra-Class Coefficient
FAS: Family Affluence Scale
HBSC: Health Behavior in School-aged Children
LR-Test: Likelihood-Ratio-Test
MVPA: Moderate-to-vigorous physical activity
PA: Physical activity
SES: Socio-economic status
US: United States
WHO: World Health Organization
